# Assessment of serum leptin, pregnancy-associated plasma protein A and CRP levels as indicators of plaque vulnerability in patients with acute coronary syndrome

**DOI:** 10.5830/CVJA-2012-008

**Published:** 2012-07

**Authors:** Moushumi Lodh, Binita Goswami, Ashok Parida, Surajeet Patra, Alpana Saxena

**Affiliations:** Department of Biochemistry, The Mission Hospital, Durgapur, West Bengal, India; Department of Biochemistry, GB Pant Hospital, New Delhi, India; Department of Cardiology, The Mission Hospital, Durgapur, West Bengal, India; Department of Biochemistry, Lady Hardinge Medical College and associated hospitals, New Delhi, India; Department of Biochemistry, Maulana Azad Medical College and associated hospitals, New Delhi, India

**Keywords:** leptin, hs-CRP, PAPP-A, metalloproteinase, plaque rupture

## Abstract

**Introduction:**

A multifactorial aetiology of coronary artery disease (CAD) has been established in the recent past. Extensive research is now underway to understand the mechanisms responsible for plaque vulnerability. The identification of a novel biomarker that will help in the assessment of plaque status is urgently needed for the purpose of patient stratification and prognostication. The aim of the present study was to evaluate leptin, pregnancy-associated plasma protein A (PAPP-A) and C-reactive protein (CRP) levels in patients with acute coronary syndrome and to assess their diagnostic efficacy in the identification of vulnerable plaques.

**Methods:**

The study group comprised 105 patients who had chest pain along with ECG changes (ST elevation, ST depression, T inversion) and raised cardiac enzyme levels. Sixty-two patients with chest pain and ECG changes but with normal cardiac enzyme profiles were included in the control group. Lipid profiles, and leptin, PAPP-A and CRP levels were assessed in these two groups. Receiver operating characteristics (ROC) curves were plotted to determine the utility of the parameters under study as markers of plaque vulnerability.

**Results:**

Significantly higher levels of serum lipoprotein (a), leptin, PAPP-A and high-sensitivity CRP (hs-CRP) were observed in the cases than in the controls. A positive correlation was observed between CRP and PAPP-A levels as well as CRP and leptin concentrations. ROC curve analysis revealed similar efficacies of CRP and PAPP-A levels in their ability to detect unstable plaques with areas under the curve of 0.762 and 0.732, respectively. Multivariate analysis established the superiority of hs-CRP as a predictor of plaque instability.

**Conclusions:**

Our study highlights the utility of both CRP and PAPP-A levels as determinants of plaque instability. Our findings necessitate population-based follow-up studies to establish the superiority of either of the two biomarkers in the field of preventive cardiology.

## Abstract

Acute coronary syndrome (ACS) accounts for 20% of all medical emergency admissions and has the highest risk for adverse effects and deaths.^1^ ACS encompasses patients who present with unstable ischaemic heart disease (ST elevation myocardial infarction, non-ST-elevation myocardial infarction). The rupture of coronary atherosclerotic plaque and subsequent thrombus formation are major events underlying ACS. Apart from traditional risk factors, several novel risk factors have been found to be associated with acute coronary syndromes.^2^

Atherosclerosis results due to the complex interplay between environmental, genetic and individual risk factors. Physical inactivity, along with the intake of calorie-dense food account for the energy imbalance that is widely prevalent in developing and developed countries. The metabolic perturbations due to this energy imbalance along with smouldering inflammatory processes and other as yet poorly understood mechanisms initiate and aggravate the atherosclerotic process.^3^,^4^

The scientific community is engrossed in extensive research in order to discover biomarkers that will help stratify patients who are more prone to develop the complications consequent to atherosclerosis. The inability of lipid levels to provide an insight into the ongoing atherosclerotic process and impending complications has further necessitated the identification of novel markers that assess the dynamics of atherosclerosis. Adipocytokines (leptin), pregnancy-associated plasma protein A (PAPP-A) and high-sensitivity C-reactive protein (hs-CRP) are the newer emerging biomarkers for assessing plaque stability.^5^

Adipose tissue is now identified as an active endocrine organ and not merely a storage site for fat. The mediators released by adipose tissue are known as adipocytokines or adipokines.^6^ Leptin is one such adipokine implicated in a number of physiological processes.^7^ Other important adipokines are adiponectin and resistin. It has been demonstrated that alterations in the plasma levels of these adipokines as well as disturbances in the signalling pathways disrupt the delicate adipokine biology and subsequently give rise to many disorders.^6^ Recent studies have indicated that hyperleptinaemia may serve as a cardiovascular risk factor.^8^-^10^

PAPP-A is a glycoprotein produced by the placental synctiotrophoblastic cells and was initially discovered in the sera of pregnant women.^11^ However, many non-placental sites of PAPP-A production have also been identified. It is a member of the metzincin metalloproteinase superfamily. Metalloproteinases may contribute to the fragility of the lipid-rich atherosclerotic plaque and eventually to its rupture by degrading the extracellular matrix.^12^ Recent studies by Bayes-Genis *et al*. reveal that it may be a potential determinant of plaque instability by degrading the extracellular matrix of the fibrous cap of the atherosclerotic plaque.^13^

C-reactive protein, determined by high-sensitivity techniques, is the most widely studied marker of inflammation in the field of atherosclerosis. Its role has been identified in the initiation, progression and final outcome of the atherosclerotic process. It is an acute-phase reactant and is an important player in the inflammatory process observed to be active in atherosclerosis. Studies have revealed that it is not an innocent bystander and indicator of the inflammatory process; rather it is actively involved in the pathogenic mechanisms underlying atherosclerosis.^14^,^15^

Lipoprotein (a) is a cardiovascular risk factor highly prevalent in subjects with Asian Indian origin. It has both thrombogenic and atherogenic properties. This is due to its homology to plasminogen and its low-density lipoprotein (LDL)-like properties. Hence it is known as a dual pathogen. It may play an important role in the susceptibility of atherosclerotic plaques to instability.^3^

The present study aimed to evaluate these novel risk factors and to look for interrelationships between them in the setting of acute coronary syndrome.

## Methods

The study was jointly conducted by the Departments of Biochemistry and Medicine, Maulana Azad Medical College and associated hospitals, New Delhi, India. The study involved screening of 379 patients with symptoms of ‘chest pain’, presenting to the medical emergency department during the period between April and October 2010. These patients were then further evaluated by electrocardiography. One hundred and sixty-seven patients demonstrated ECG abnormalities such as ST elevation, ST depression and T inversion. They were enrolled as the study population.

Cardiac enzymes such as troponins and CK-MB levels were evaluated in these patients. One hundred and five patients had raised serum cardiac enzyme concentrations and hence depicted the onset of acute coronary syndrome. These patients were classified as cases and they were further evaluated by coronary angiography.

Angiography was conducted by cardiologists using standard techniques. The angiograms were done by two different cardiologists who did not have any information regarding patient identity and aetiology, to avoid any subjective bias. Those who had normal cardiac enzyme concentrations were enrolled as controls.

The inclusion criteria were:

• AMI patients diagnosed by typical rise and gradual fall of biochemical markers (troponins, CK-MB) of myocardial necrosis with at least one of the following:
- ischaemic symptoms
- development of pathological Q waves on the ECG reading
- ECG changes indicative of ischaemia (ST elevation or depression)
- coronary artery intervention (e.g. coronary angioplasty)
• Unstable angina: chest discomfort at rest with either ST depression of at least 0.1 mV or T-wave inversion in two or more contiguous ECG leads, CK-MB levels normal and angiographically confirmed coronary artery disease (CAD).

The exclusion criteria that were considered during the selection of the study population were:

• advanced kidney failure indicated by high serum urea and creatinine
• overt heart failure as diagnosed by echocardiography
• history of major surgery/trauma within the previous month
• suspected systemic inflammatory diseases
• pregnancy
• chronic stable angina (effort induced) diagnosed as chest pain of at least six months’ duration, accompanied by severe CAD on angiography (70% stenosis in any major artery).

A fasting blood sample (8–10 ml) was drawn prior to angiography and the serum was extracted and stored at –70°C until further testing. Fasting serum leptin, hs-CRP and PAPP-A levels were estimated with ELISA kits provided by DRG International, Germany.

## Statistical analysis

The data were expressed as mean ± standard deviation. Student’s *t*-test was used to compare the values between the cases and controls. Spearman’s correlation analysis was used to find the association between the various parameters of our study. A *p*-value < 0.05 was accepted as statistically significant. Multivariate regression analysis was carried out to assess the role of the different biomarkers in unstable CAD. All statistical analyses were performed with the program Statistical Package for the Social Science 12.0 (SPSS Inc, Chicago, Illinois).

## Results

The demographic and risk-factor profiles of the study population are shown in Table 1. One hundred and five patients fulfilled the inclusion criteria for enrolment as cases (acute myocardial infarction) while 62 were classified as controls (angina pectoris). The mean age of the cases was higher than the controls (56.9 ± 11.1 vs 48.8 ± 10.9 years, respectively). Females comprised 18% of the cases and 23% of the controls.

**Table 1.** Demographic Details And Risk Factor Profile Of The Study Population

*Characteristics*	*Cases (n = 105)*	*Controls (n = 62)*	p*-value*
Age (years)	56.9 ± 11.1	48.8 ± 10.9	NS
Male/female	86/19	45/17	–
BMI (kg/m^2^)	28.5 ± 5.7	25.2 ± 5.3	–
Waist circumference (cm)	101.46 ± 10.2	88.9 ± 15.9	< 0.01
Smoking (%)	50 (48%)	9 (13%)	< 0.01
Hypertension (%) or antihypertensive drugs taken	55 (52%)	31 (50%)	NS
Diabetes (%) or hypoglycaemic drugs	46 (44%)	4 (8%)	< 0.001
Hypolipidaemic drugs	42 (40%)	15 (25%)	< 0.01

The body mass index and waist circumferences were higher in the cases compared to the controls. The prevalence of smoking and diabetes mellitus was significantly higher in the cases than the the controls. Subjects with none of the above risk factors comprised 16% of the cases and 28% of the controls, respectively.

The biochemical profile of the study population is given in Table 2. Total cholesterol, triglyceride and LDL cholesterol levels were higher in the patients compared to the controls (167.7 ± 32.2 vs 150.1 ± 43. 9 mg/dl; 136.9 ± 61.7 vs 125.2 ± 49.6 mg/dl; 112.7 ± 29.8 vs 100.5 ± 27.3 mg/dl, respectively). However the differences were not statistically significant.

**Table 2.** Biochemical Profile Of The Study Population

*Characteristics*	*Cases*	*Controls*	p*-value*
Total cholesterol (mg/dl)	167.7 ± 32.2	150.1 ± 43. 9	0.063
Triglycerides (mg/dl)	136.9 ± 61.7	125.2 ± 49.6	0.353
LDL-C (mg/dl)	112.7 ± 29.8	100.5 ± 27.3	0.067
HDL-C (mg/dl)	30.6 ± 6.8	38.8 ± 7.6	< 0.001
Lipoprotein (a) (mg/dl)	34.8 ± 30.1	16.7 ± 13.5	< 0.001
Leptin (ng/ml)	11.97 ± 8.5	8.06 ± 5.8	0.01
PAPP-A (mIU/l)	16.84 ± 8.93	10.4 ± 5.8	< 0.001
Hs-CRP (mg/l)	5.46 ± 2.8	2.93 ± 2.49	< 0.001

High-density lipoprotein (HDL) levels were significantly lower in the cases than the controls (30.6 ± 6.8 vs 38.8 ± 7.6 mg/dl; *p* < 0.001). Lipoprotein (a) [lp(a)] levels demonstrated a non-Gaussian distribution pattern with significantly higher levels (34.8 ± 30.mg/dl) in the cases compared to 16.7 ± 13.5 mg/dl in the controls (*p* < 0.001). Significantly higher levels of serum leptin, PAPP-A and hs-CRP were observed in the cases compared to the controls (11.97 ± 8.5 vs 8.06 ± 5.8 ng/ml; 16.84 ± 8.93 vs 10.4 ± 5.8 mIU/l and 5.46 ± 2.8 vs 2.93 ± 2.49 mg/l, respectively).

In order to evaluate the performance of serum lp(a), leptin, PAPP-A and hs-CRP levels as indicators of plaque rupture, the receiver operating characteristics (ROC) curves were plotted. Table 3 illustrates the findings elaborated by the ROC curves. The area under the curve for hs-CRP was highest at 0.762, followed closely by PAPP-A at 0.732. Cut-off points of these parameters were determined from the curves, which could help in prospective stratification of patients predisposed to unstable atherosclerotic sequelae.

**Table 3.** Receiver Operating Characteristics Of The Parameters Under Study

*Characteristics*	*AUC*	p*-value*	*Cut offs chosen from ROC curves*
Lipoprotein (a)	0.778	0.001	15.9 mg/dl
			Sensitivity 72%
			Specificity 70%
Leptin	0.705	0.015	6.9 ng/ml
			Sensitivity 66%
			Specificity 60%
PAPP-A	0.901	0.002	11.75 mIU/l
			Sensitivity 68%
			Specificity 60%
Hs-CRP	0.849	< 0.001	3.1 mg/l
			Sensitivity 77%
			Specificity 73%

**Fig. 1.**
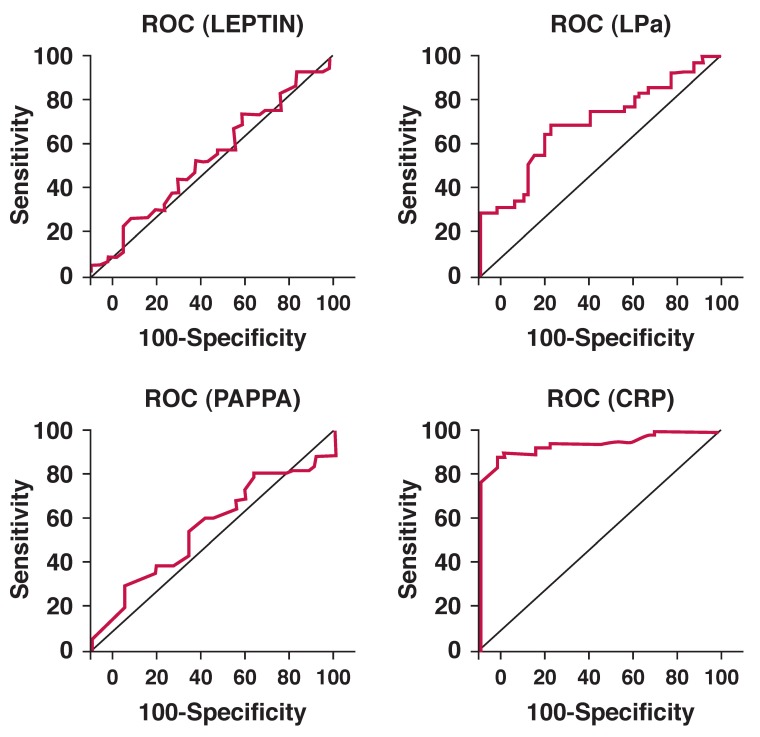
ROC curves of the different parameters under study.

According to this, a cut off of 3.1 mg/l for hs-CRP has a sensitivity of 77% and specificity of 73%. Similarly, a watershed value of 11.75 mIU/l for PAPP-A exhibited 68% sensitivity and 60% specificity. An attempt to increase the sensitivity was accompanied by a corresponding reduction in specificity and vice versa. Our study therefore proves the superiority of hs-CRP as a marker of plaque rupture. However, PAPP-A also proved to be a sensitive marker. Fig. 1 illustrates the ROC curves of the different parameters in the study.

A positive correlation was observed between hs-CRP with PAPP-A levels (*r* = 0.658, *p* < 0.01) and leptin (*r* = 0.612, *p* < 0.01). However, no such correlations were observed between leptin, PAPP-A and lipid levels. This finding highlights the interaction between hs-CRP and PAPP-A in initiating plaque rupture. Multivariate regression analysis proved the superiority of CRP over all the other parameters, with the order of significance being CRP > CK-MB > HDL > total cholesterol/HDL ratio > LDL/HDL ratio > fasting plasma glucose > lp(a) > total cholesterol > LDL > insulin > triglycerides > PAPP-A > leptin (Table 4).

**Table 4.** Multivariate Regression Analysis Of The Various Parameters In The Study

Fasting plasma glucose	0.194	Triglycerides	0.010
Fasting insulin	0.026	Cholesterol	0.051
Leptin	0.000	LDL	0.041
CK-MB	0.383	HDL	0.241
PAPP-A	0.001	LDL/HDL	0.202
CRP	0.396	Cholesterol/HDL	0.230
Lipoprotein (a)	0.111

## Discussion

The burden of atherosclerotic disease is a major public health problem in developed as well as developing countries and is primarily attributed to physical inactivity, calorie-rich food and a host of environmental and individualistic factors.^16^ The present study was undertaken to determine the efficacy of the novel biomarkers, adipocytokines (leptin), inflammatory mediator (hs-CRP) and a metalloproteinase (PAPP-A), for the identification of unstable plaques.

In order to achieve the goal of our study, the patients presenting to the hospital with acute coronary syndrome were classified as cases or controls depending on the angiographic findings. Those patients with normal coronary arteries comprised the control group and those with documented lesions in their coronary arteries were included in the patient group. The novelty of our study lies in the comparison of four markers of cardiovascular risk: dyslipidaemia [lp(a)], insulin reistance (leptin), inflammation (hs-CRP) and matrix remodelling (PAPP-A), as possible predictors of plaque instability. This holistic view of plaque instability was the cardinal point of our study.

Our study revealed that serum leptin levels were significantly higher in the patient group than in the controls. Hyperleptinaemia has been implicated in the aetiopathogenesis of atherosclerosis in a number of studies.^17^-^20^ The various underlying mechanisms responsible for its pro-atherogenecity are being revealed with a multitude of *in vitro* and *in vivo* studies. These include influences on cytokine signalling mechanisms,^21^ stimulation of pro-inflammatory cytokine production,^22^ stimulation of smooth muscle cell proliferation,^23^ up regulation of phagocytotic mechanisms,^17^ platelet aggregation and thrombosis,^24^,^25^ and the promotion of endothelial dysfunction.^26^

Serum leptin is also responsible for the stimulation of mitochondrial superoxide production^27^ and calcification of smooth muscle cells.^21^ It has been observed that leptin levels correlate with insulin resistance, obesity and cardiovascular risk factors. In fact, it has been hypothesised that the disturbances in adipocytokine balance and signalling pathways in obesity eventually culminate in atherosclerotic disease.^23^,^28^

Our findings are in accordance with studies carried out by many researchers. Serum leptin levels could predict adverse cardiovascular events, even after adjustment for the traditional risk factors, in the WOSCOPS trial.^18^ Cincone *et al*. reported a positive correlation between serum leptin levels and intima–media thickness in healthy individuals.^29^ Karaduman *et al*. evaluated the levels of hs-CRP and leptin in atherosclerotic plaques and found leptin levels to be significantly higher in diabetics than non-diabetics.^17^

Iribarren *et al*. reported a positive correlation between serum leptin levels and coronary artery calcium scores in healthy women.^30^ Reilly *et al*. concluded from their study that plasma leptin levels may represent a marker for adiposity, insulin resistance and vascular dysfunction, leading eventually to atherosclerosis.^31^ However, the Quebec Heart study as well as a study carried out by van den Beld did not find any correlation between leptin levels and cardiovascular events.^32^

C-reactive protein level has been established as a determinant of the inflammatory process in atherosclerosis. In fact, hs-CRP levels have been correlated with disease initiation, progression and prognosis.^33^ CRP carries out a multipronged attack on the endothelium with pro-inflammatory, stimulatory and pro-oxidant actions leading to endothelial dysfunction and other events that predispose to atherosclerosis. The mechanisms are explained in our previous article.^34^

The present study also highlights the significance of hs-CRP levels in determining CAD. The ability of statins to decrease CRP levels has garnered interest in the recent years. The PROVE IT TIMI 22 (PRavastatin Or atorVastatin Evaluation and Infection Therapy – Thrombolysis In Myocardial Infarction 22) study highlighted that the CRP levels after statin therapy were as efficient in predicting CAD as LDL levels.^35^ The recent JUPITER study also highlighted the pro-atherosclerotic role of CRP and its response to statin therapy.^36^

Pravastatin therapy led to maximal benefit in patients with highest CRP values regardless of their LDL concentrations, according to the Cholesterol And Recurrent Events (CARE) trial.^37^ Similar findings were reported by the AFCAPS/TexCAPS24 and Physicians Health study.^38^,^39^ Our study also highlights the importance of hs-CRP in the prediction of unstable plaques. However, caution is warranted in the interpretation of the utility of hs-CRP due to its non-specificity. Being an acute-phase reactant, its significance must be analysed by excluding all other causes of its elevation.

Anand *et al*. concluded from their study that CRP was independently associated with CAD in Asian Indians after adjustment for Framingham risk factors and anthropometric measurements.^40^ The INTERHEART study demonstrated that increased apolipoprotein B/apolipoprotein AI, smoking, abdominal obesity, psychosocial stress, diabetes mellitus and hypertension accounted for 90% of the CAD risk in Asian Indians.^41^

PAPP-A is an emergent biomarker signifying plaque instability. The role of PAPP-A as a biomarker for unstable atherosclerotic plaques was initially suggested by Bayes-Genis *et al*., who measured PAPP-A levels in the cells and the extracellular matrix of unstable plaques in eight patients who had sudden death due to cardiac causes.^13^

PAPP-A is a member of the metzincin metalloproteinase superfamily and is also produced by non-placental tissue such as fibroblasts, vascular endothelial cells and vascular smooth muscle cells. The circulating form of the protein comprises a heterotetrameric complex formed of two subunits of 200 and 250 kDa, bound by eosinophil major basic protein.^42^ PAPP-A is secreted by activated macrophages that take part in the atherosclerotic process.

Studies have revealed that PAPP-A contributes to plaque instability by promoting degradation of the extracellular matrix of the fibrous cap.^43^ PAPP-A acts as an insulin-like growth factor (IGF) binding protein 4 protease. PAPP-A degrades IGF-binding protein 4 protease and thus increases the availability of free IGF-I.^44^ IGF-I induces macrophage activation, chemotaxis, LDL cholesterol uptake by macrophages, and the release of pro-inflammatory cytokines by these cells.^45^,^46^ IGF-I is also implicated in the proliferation and migration of smooth muscle cells and the consequent vascular response to injury.^45^,^47^ These events lead to the initiation of plaque destabilisation.

We observed significantly higher levels of PAPP-A in the patients compared to the control group. It also emerged as a good indicator of plaque vulnerability, just behind hs-CRP, with an area under curve of 0.732. Our findings corroborate the results of previous studies that have been carried out.^48^,^49^ Lund *et al*. concluded that PAPP-A was a good predictor of both ischaemic cardiac events and the need for revascularisation in patients with ACS.^50^ Cosin-Sales *et al*. concluded from their study on patients with ACS that those with complex coronary lesions had significantly higher PAPP-A levels than patients who were free of such lesions.^13^ Heeschen *et al*. evaluated PAPP-A levels in 547 patients with angiographically proven ACS. They found that elevated PAPP-A levels indicated an increased risk of plaque rupture, myocardial infarction, revascularisation and cardiovascular death in both troponin T-positive or -negative patients.^51^

Studies in patients with stable angina have proved the superiority of PAPP-A over troponin T and hs-CRP levels.^52^ Elevated PAPP-A levels in asymptomatic hyperlipidaemic patients were associated with increased echogenicity of carotid atherosclerotic plaques.^53^ Experimental as well as epidemiological data substantiated the role of PAPP-A in the detection of unstable CAD in patients with normal troponin levels.^54^,^55^ This could help in the prompt identification and timely intervention in high-risk populations.

We also observed a statistically significant correlation between PAPP-A and CRP levels in patients with ACS. It has been reported that pro-inflammatory cytokines such as TNF-α stimulate PAPP-A synthesis by macrophages. The accumulation of activated macrophages at the site of unstable plaques leads to the release of PAPP-A by these cells.^56^ The correlation between these two mediators suggests the complex interplay between various pathways that eventually converge to promote plaque instability. Such an association between CRP and PAPP-A levels was also reported by Heeschen *et al*.^51^ and Jin-Lai *et al*.^57^

A similar correlation was observed between CRP and leptin levels. Obesity is associated with an inflammatory response characterised by high levels of pro-inflammatory cytokines.^58^ Receptors for leptin and cytokines are structurally related. Leptin can also directly induce the production IL-6, which is the most potent stimulator of CRP synthesis.^59^ Hence a positive correlation was observed between leptin and CRP levels. Obesity therefore leads to a higher risk of CAD due to the synergism between hyperleptinaemia and the on-going low-grade inflammatory process.

## Conclusions

Coronary artery disease is the root cause of mortalities due to non-infectious aetiology. The initiation of florid plaque rupture and the consequent complications lead to various life-threatening sequelae of atherosclerotic disease. Hence, identification of plaque vulnerability at the incipient stages may help in effective and timely management.

Currently, detection of plaque status involves invasive procedures such as coronary angiography and intravascular ultrasound, which have their own shortcomings due to their invasive nature and patient non-compliance, as well as financial constraints. Therefore the identification of a simple blood test that may aid in this endeavour is the need of the hour.

We observed hs-CRP and PAPP-A levels to be effective indicators of plaque rupture. Interpretation of CRP data requires consideration of confounding factors such as inflammation.

PAPP-A levels are not affected by infections or any underlying inflammation. Therefore assessment of PAPP-A levels may be a very attractive alternative for detecting unstable plaque activity and hence useful in stratification and prognostication of subjects. The ability of PAPP-A to detect plaque function even when the other markers of myocardial injury such as troponins are not elevated, is another justification for evaluation of PAPP-A level as a novel marker of plaque vulnerability. The comparison of PAPP-A and hs-CRP levels with the established biomarker troponin T needs to be evaluated and the results substantiated by studies carried out in large, population-based studies.
